# Comparison of the efficacy and safety between dezocine injection and morphine injection for persistence of pain in Chinese cancer patients: a meta-analysis

**DOI:** 10.1042/BSR20170243

**Published:** 2017-06-07

**Authors:** Lei Wang, Xudong Liu, Jianfeng Wang, Yingjiao Sun, Guozhuan Zhang, Lishuang Liang

**Affiliations:** Department of Pain, Qilu Hospital of Shandong University, Ji’nan, Shandong, P. R. China

**Keywords:** Cancer pain, Dezocine Injection, Efficacy, Morphine Injection, Meta-analysis, Safety

## Abstract

The association between the efficacy and safety of dezocine injection and morphine injection for persistence of pain in patients with cancer had yielded controversial results. Therefore, we conduct a meta-analysis of existing observational published studies to assess the relationship between them among Chinese. We conducted a comprehensive research from the databases of PubMed, Web of Science, and Wan Fang Med Online for the related studies up to October 2016. Summary odds ratio (OR) with 95% confidence interval (95% CI) were calculated with the random effects model. Nine published studies comprising 333 dezocine injection patients and 321 morphine injection patients were included in this meta-analysis. Our results suggested that there was no statistical significance between dezocine injection and morphine injection at the case number of effective pain relief (EPR) [OR = 0.97, 95% CI (0.77–1.22), *I*^2^ = 0.0, *P* for heterogeneity = 1.000]. However, the rate of adverse drug reaction (ADR) caused by dezocine injection was 56% less than that caused by morphine injection, the difference was statistically significant [OR = 0.44, 95% CI (0.30–0.65), *I*^2^ = 0.0, *P* for heterogeneity = 0.980]. No between-study heterogeneity and publication bias were found. In conclusions, this meta-analysis indicates that there is no significant association on the efficacy of persistence of pain in patients with cancer between dezocine injection and morphine injection among Chinese. However, dezocine injection was with less ADR compared with morphine injection.

## Introduction

In recent years, with the development of medical technology, the survival of cancer patients has been significantly extended, and the life quality of cancer patients had been caused more and more attention. Pain is one of the most common and most intolerable symptoms of cancer patients and a major factor in the quality of life. According to the World Health Organization (WHO) statistics, at least one-third of cancer patients with varying degrees of pain, advanced patients can be as high as 60 to 90% [1].

Dezocine, as a representative opioid-receptor agonist/antagonist, has been used widely in clinical practice [2,3]. A previous publication found that dezocine was a promising and safe analgesic that was slightly more potent than morphine for the relief of perioperative pain [4]. However, another paper reported that dezocine (10 mg) reportedly appeared equipotent with morphine (10 mg) [5]. So, the association between the dezocine injection and morphine injection for persistence of pain in patients with cancer has yielded controversial results. In the present study, we conducted a meta-analysis to assess if dezocine injection was more effective and safe for persistence of pain in patients with cancer compared with morphine injection.

## Methods

### Literature search strategy and inclusion criteria

We conducted a literature search to identify relevant available articles published from the databases of PubMed, Web of Science, and Wan Fang Med Online up to October 2016. Search terms included ‘dezocine’ AND ‘morphine’ AND ‘injection’ AND (‘efficacy’ OR ‘safety’) AND (‘China’ or ‘Chinese’). We also reviewed the reference lists of the included studies for those undetected relevant studies. We used the guidelines of the ‘Preferred Reporting Items for Systematic Reviews and Meta-Analyses (PRISMA)’ [6] to perform the study selection process.

All relevant studies reporting the efficacy or safety between dezocine injection and morphine injection for persistence of pain in patients with cancer among Chinese were considered for inclusion. The inclusion criteria were as follows: (1) the study type was cohort, case–control, randomized controlled trial, or cross-sectional design; (2) the exposure of interest was using dezocine injection or morphine injection for persistence of pain in patients with cancer; (3) the outcome of interest was the case number of effective pain relief (EPR) or the numbers of adverse drug reaction (ADR); (4) the study reported the country in China; (5) the study should be written in English or Chinese. Accordingly, the studies were excluded if: (1) they were review articles; (2) they repeated or overlapped publications; (3) the report was on animal study; (4) the study reported in other countries.

### Data extraction

Two researchers independently reviewed the suitable studies. The data extracted from the studies in use are referring such aspects: name of the first author, year of publication, age for subjects, the ways of treating cancer patients for persistent pain, sample size for the case number of EPR or ADR and participants, and odds ratio (OR) with 95% confidence intervals (CI) reporting the efficacy or safety between dezocine injection and morphine injection for persistent pain in cancer patients, if available. Disagreement between the two investigators will be resolved by consensus with a third reviewer.

### Statistical analysis

We analyzed dichotomous variables by estimating OR with their 95% CI of the efficacy or safety between dezocine injection and morphine injection for persistence of pain in patients with cancer. A random-effects model was used to combine the whole pooled effect, which considers both within-study and between-study variation [7]. The statistical heterogeneity was analyzed using Cochran *I*^2^, and *I*^2^ values of 0, 25, 50 and 75% represent no, low, moderate, and high heterogeneity, which depicts the percentage of variation across studies due to heterogeneity rather than chance [8,9]. All of the Egger’s test [10] and funnel plot [11] were used to evaluate the publication bias. Sensitivity analysis [12] was performed to look for the difference of summary RR with 95% CI when removing an individual studied at a time. STATA version 10.0 was used for the whole meta-analysis. Statistical significance was set at *P*<0.05.

## Results

### Articles selection

**Figure 1:**
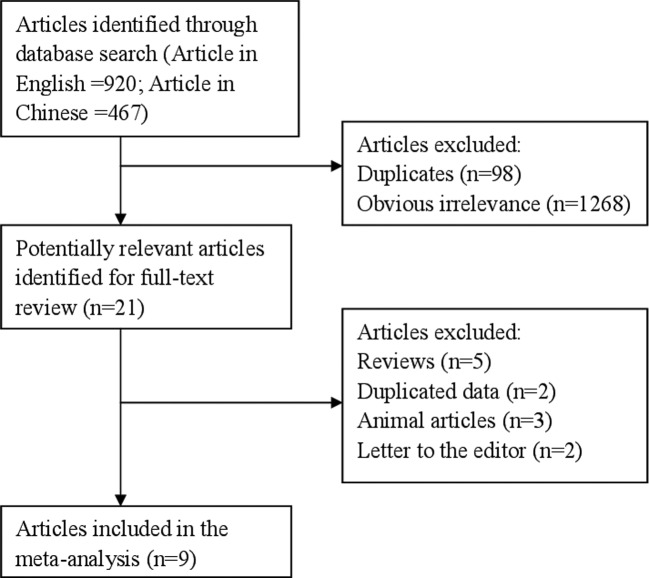
The flow diagram of screened, excluded, and analyzed publications.

The number of articles searched from the databases of PubMed, Web of Science, and Wan Fang Med Online was 1387 (articles in English = 920; articles in Chinese = 467). Ninety-eight articles were duplicates from the databases of PubMed and Web of Science. When we reviewed the title and abstract, 1268 articles were further excluded due to obvious irrelevance. The remaining 21 articles were reviewed thoroughly. However, 5 review articles, 2 duplicates data, 3 animal articles, and 2 letters to the editors were excluded once again. Therefore, 9 articles [13–21] were included in this report at the end. Figure 1 shows the study flowchart of this meta-analysis. The characteristics of included article are showed in Table 1.

**Table 1:** Characteristics and methodological quality of included studies

Study (author, year)	Study design	Dezocine injection group				Morphine injection group				Adverse events type
		Age	Effective	Events	Patients	Age	Effective	Events	Patients	
Qi et al. (2011)	RCT	57.7 ± 6.7	27	2	28	57 ± 8.6	25	5	25	Dizziness, nausea, vomiting
Zhang et al. (2012)	RCT	56.2 ± 3.2	38	5	40	56.4 ± 2.3	39	13	40	Dizziness, nausea, vomiting
Li et al. (2013)	RCT	57.4 ± 6.6	26	1	30	57.1 ± 8.5	25	4	27	Dizziness, nausea, vomiting
Shen et al. (2013)	RCT	53 ± 9	34	5	36	53 ± 10	33	13	36	Dizziness, nausea, vomiting, poor appetite
Li et al. (2014)	RCT	67 ± 6	60	24	62	69 ± 6	61	50	62	Dizziness, nausea, vomiting, sleepiness
Yao et al. (2015)	RCT	NA	30	6	40	NA	31	8	40	NA
Shi et al. (2013)	RCT	58.5 ± 8.3	41	5	43	58.5 ± 8.3	39	14	43	Dizziness, nausea, vomiting
Gan et al. (2012)	RCT	NA	20	NA	23	NA	21	NA	23	NA
Sun et al. (2014)	RCT	58.2 ± 9.0	NA	2	31	58.5 ± 15.1	NA	4	25	Dizziness, nausea, vomiting

### Comparison of the efficacy between dezocine injection and morphine injection

**Figure 2:**
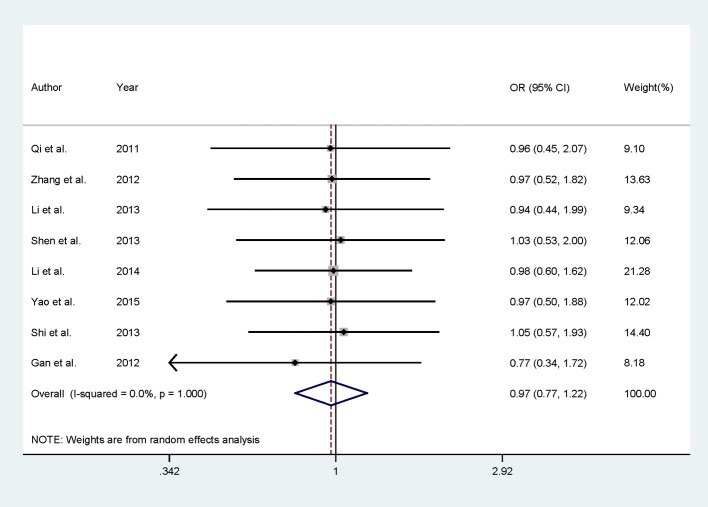
Comparison of dezocine injection and morphine injection for persistence of pain in Chinese patients with cancer.

Eight published studies reported the efficacy between dezocine injection and morphine injection comprising 302 dezocine injection patients, and 296 morphine injection patients were included in this meta-analysis. In the summary result, we did not find any significant association between dezocine injection and morphine injection for the efficacy of persistence of pain in patients with cancer among Chinese populations [RR = 0.97, 95% CI (0.77–1.22)] (Figure 2). We also did not find any between-study heterogeneities [*I*^2^ = 0.0, *P* for heterogeneity = 1.000] in the overall result.

### Comparison of the safety between dezocine injection and morphine injection

**Figure 3:**
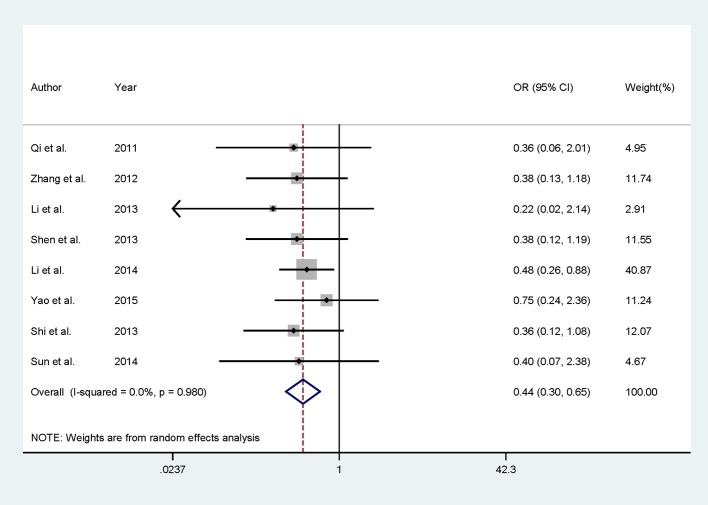
Comparison of dezocine injection and morphine injection with respect to complications.

All of the included studies except Gan et al. [20] involving 310 dezocine injection patients and 298 morphine injection patients assessed the ADR of dezocine injection and morphine injection. Seven of the included studies indicated that there is no significant association for the safety of dezocine injection group compared with morphine injection group. Only one study suggested that dezocine injection group had lower ADR than morphine injection group. Summary results showed significantly lower ADR in dezocine injection group compared with morphine injection group [RR = 0.44, 95% CI (0.30–0.65)], with no between-study heterogeneity was found [*I*^2^ = 0.0, *P* for heterogeneity = 0.980] (Figure 3).

### Sensitivity analysis and publication bias

**Figure 4:**
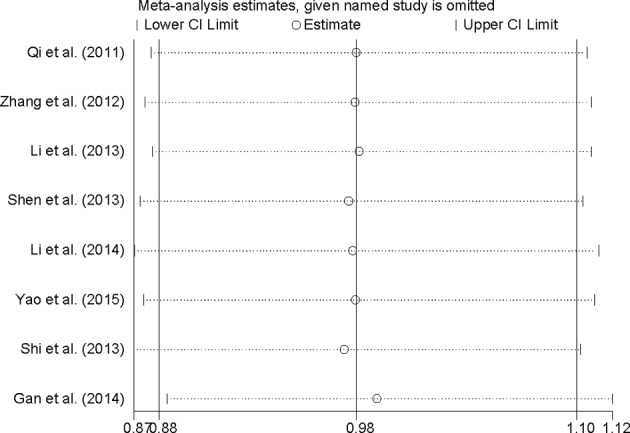
Analysis of influence of individual study for persistence of pain in patients with cancer, comparison of dezocine injection and morphine injection. Open circle, the pooled OR, given named study is omitted. Horizontal lines represent the 95% CIs.

**Figure 5:**
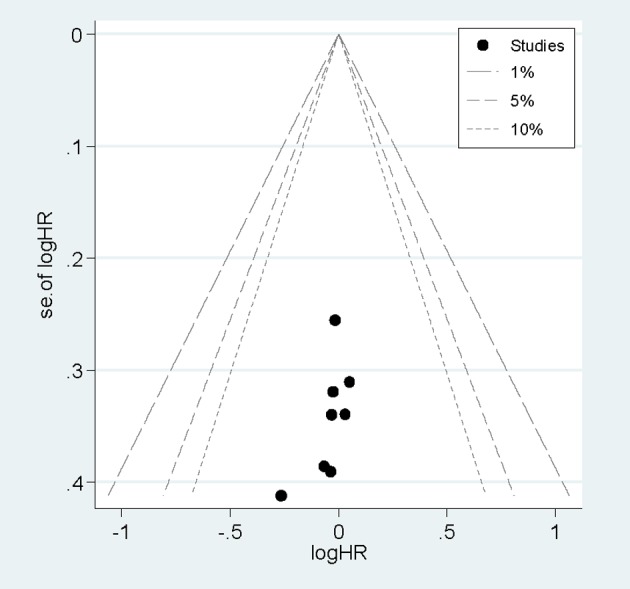
Funnel plots of dezocine injection and morphine injection for persistence of pain in patients with cancer. Funnel plots of dezocine injection and morphine injection for persistence of pain in patients with cancer.

Sensitivity analysis showed that there is no individual study that had excessive influence on summary result when removing one study at a time for the efficacy of dezocine injection and morphine injection for persistence of pain in patients with cancer (Figure 4). Egger’s test (*P*=0.143) and funnel plot (Figure 5) showed no evidence of significant publication bias between dezocine injection group and morphine injection group for persistence of pain in patients with cancer.

## Discussion

Because of the limited level of medical development, many cancer patients in the diagnosis have gone beyond the scope of radical treatment. Cancer pain in cancer patients, especially in advanced cancer patients with common symptoms, is one of the most intolerable symptoms. Cancer pain could be the cause of destruction of the quality of life of patients from the physical, psychological, spiritual, and social aspects, giving a great pain to the patient. Therefore, we conducted this meta-analysis to assess if dezocine injection was more effective and safe for persistence of pain in patients with cancer compared with morphine injection. Findings from this report indicated that there is no significant association on the efficacy for persistence of pain in patients with cancer between dezocine injection and morphine injection when summarizing the result for EPR. However, dezocine injection was significantly with less ADR compared with morphine injection. We did not find any between-study heterogeneity and publication bias during Cochran *I*^2^ test, Egger’s test, and funnel plot.

Previous report had suggested that combinatory use of dezocine and midazolam in addition to local anesthetic in retrobulbar nerve block can help relief pain and anxiety during vitrectomy and reduce postoperative nausea and vomiting [22]. Another study among health mice indicated that dezocine antagonizes morphine analgesia on acute nociception upon simultaneous administration [23]. A study conducted by the Xu et al. [24] showed that dezocine combined with propofol can be successfully used for the anesthetization of indolent colonoscopy patients without pain and should be widely used. Therefore, our meta-analysis obtained a consistent result with the above-mentioned studies. The difference with those studies is that dezocine injection was more safe compared with morphine injection for persistence of pain in patients with cancer.

One of the advantages reported in our meta-analysis was that we included more studies to achieve a much greater possibility of reaching reasonable conclusions, during small study effect in the individual article could affect the result. Second, we did not find any significant association between dezocine injection and morphine injection on the efficacy for persistence of pain in patients with cancer, but we found that dezocine injection is more safe than morphine injection. Although only one study suggested that dezocine injection group had lower ADR that morphine injection group, while seven of the included studies indicated that there is no significant association for the safety of dezocine injection group compared with morphine injection group. More studies with large cases and participants will obtain more exact result. Third, we only analyze the studies conducted in China, this will exclude the influence by geographic locations. Furthermore, although a study [25] had indicated that between-study heterogeneity is common in the meta-analysis, no between-study heterogeneity was found in the whole or in the part analyses. The publication bias was not found among Egger’s test and funnel plot. These two factors suggested that our result was stable.

However, some limitations in our study should be concerned. First, other unpublished literatures on relevant pharmaceutical websites were not searched and only studies in English or Chinese were included, which may lead to a potential publication bias, but no significant publication bias was found by Egger’s test and funnel plot. Second, almost all the studies reported the association for efficacy and safety of dezocine injection and morphine injection for persistence of pain in patients with cancer are from China. Therefore, the result are applicable to the Chinese populations, but not be extended to other populations.

In summary, this report suggested that there is no significant association on the efficacy of persistence of pain in patients with cancer between dezocine injection and morphine injection in Chinese. However, the ADR in dezocine injection was less compared with morphine injection.
